# Effects of transcutaneous vagus nerve stimulation on motor function in older adults: a randomized crossover study

**DOI:** 10.1016/j.clinsp.2026.101141

**Published:** 2026-10-06

**Authors:** Silvia Ataides Alves Santana, Fernando Zanela da Silva Arêas, Sandy Daniely Alves Santana, Taize Iolanda de Souza Ferreira, Maycon Pelosato Duarte, Francisdeise Suave Santos, Leila Dutra de Oliveira, Cassia da Luz Goulart, Fernando Fonseca de Almeida Val, Guilherme Peixoto Tinoco Areas

**Affiliations:** a Physiology Science Laboratory, Universidade Federal do Amazonas, Manaus, AM, Brazil; b Baylor Scott and White Research Institute, Dallas, TX, USA; c Laboratory of Neuromodulation and Neurorehabilitation, Universidade Federal do Espírito Santo, Vitória, ES, Brazil; d Tropical Medicine Foundation Dr. Heitor Vieira Dourado, Manaus, AM, Brazil

**Keywords:** Neuromodulation, 6MWT, Elderly

## Abstract

**Background:**

Aging is associated with declines in gait performance and autonomic regulation, increasing the risk of functional limitations. Auricular transcutaneous Vagus Nerve Stimulation (tVNS) is a promising non-pharmacological intervention, but its acute effects on gait in older adults remain unclear. This study investigates whether acute tVNS can improve gait performance and autonomic function in healthy community-dwelling older women.

**Methods:**

A prospective, randomized, blinded, crossover trial was conducted with 17 healthy older women (≥60-years). Participants received active tVNS (left ear, 25 Hz, 500 μs, 30-min) and sham stimulation (right earlobe), separated by a 24-hour washout period. The primary outcome was the distance covered on the 6-Minute Walk Test (6MWT). Heart Rate Variability (HRV) was assessed as a key secondary outcome.

**Findings:**

Active tVNS significantly increased the resting Inter-Beat Interval (IBI, p = 0.008) and reduced heart rate (p = 0.03), indicating enhanced parasympathetic modulation. Sympathetic activity (LF and LF/HF ratio) decreased following active stimulation. Although absolute 6MWT distance did not significantly change (p = 0.07), the predicted walking distance improved significantly with active tVNS compared to sham (p < 0.01; Cohen’s *d* = 0.65; mean difference -7.2%). Other clinical parameters remained unchanged.

**Interpretation:**

This first randomized crossover trial investigating the acute effects of transcutaneous vagus nerve stimulation in healthy older women demonstrated enhanced parasympathetic modulation and improved performance relative to predicted walking capacity. However, the primary outcome, absolute 6-minute walk distance, did not reach statistical significance. These findings should therefore be considered preliminary and hypothesis-generating, supporting the need for larger trials to determine the long-term clinical relevance of tVNS in older populations.

## Highlights

• Acute tVNS increases parasympathetic function in healthy older women.
• Vagal stimulation improved predicted walking capacity in the study.
• Active tVNS led to a significant increase in resting inter-beat interval.
• tVNS is a promising non-invasive tool for aging-related functional health.

## Introduction

Transcutaneous auricular Vagus Nerve Stimulation (tVNS) is a safe and well-tolerated technique that has been reported to influence vagal regulation across several peripheral systems, including the cardiac, gastrointestinal, and immune systems.^1^, ^2^, ^3^ This modality represents an expanding field of interest due to its non-invasive nature and potential applicability to diverse clinical populations.

A landmark study by Dawson et al.^4^ reported that invasive vagus nerve stimulation in patients with severe upper limb motor impairment, nine months after stroke, facilitated motor recovery in the stimulated group. Similarly, the pilot study by Redgrave et al.^5^ demonstrated that non-invasive vagus nerve stimulation improved motor capacity in individuals with upper limb deficits secondary to central nervous system lesions. Together, these findings suggest that, in addition to invasive approaches, non-invasive stimulation may also positively influence motor performance.

The mechanistic plausibility of tVNS-induced motor effects in healthy volunteers has been linked to increased motor cortical excitability, as evidenced by evoked potentials, combined with enhanced GABAergic inhibition and reduced glutamatergic activity.^6^ To our knowledge, this is the first randomized crossover study to investigate the acute effects of transcutaneous vagus nerve stimulation on predicted functional walking capacity in healthy community-dwelling older women. Although the inclusion of only women improves sample homogeneity, it may limit the generalizability of the findings to older men. Building on this rationale, we aimed to investigate whether tVNS, when applied to community-dwelling older adults without known motor disorders, could acutely improve functional capacity compared to sham stimulation. We hypothesized that tVNS would increase the distance walked by older participants relative to the sham condition in a blinded crossover study.

## Methods

### Study design

A prospective, randomized controlled, blinded experimental crossover trial with concealed allocation, blind assessors, participants, and intention-to-treat analysis was carried out. Community-dwelling elderly women were recruited from the general community. Seventeen volunteers were enrolled in a single-center study via advertisements and pre-screening. Participants received both interventions (sham and tVNS), with a 24-hour washout period between each intervention in a crossover design. Only community-dwelling elderly women were selected to ensure homogeneous results. (Fig. 1).

**Fig. 1:**
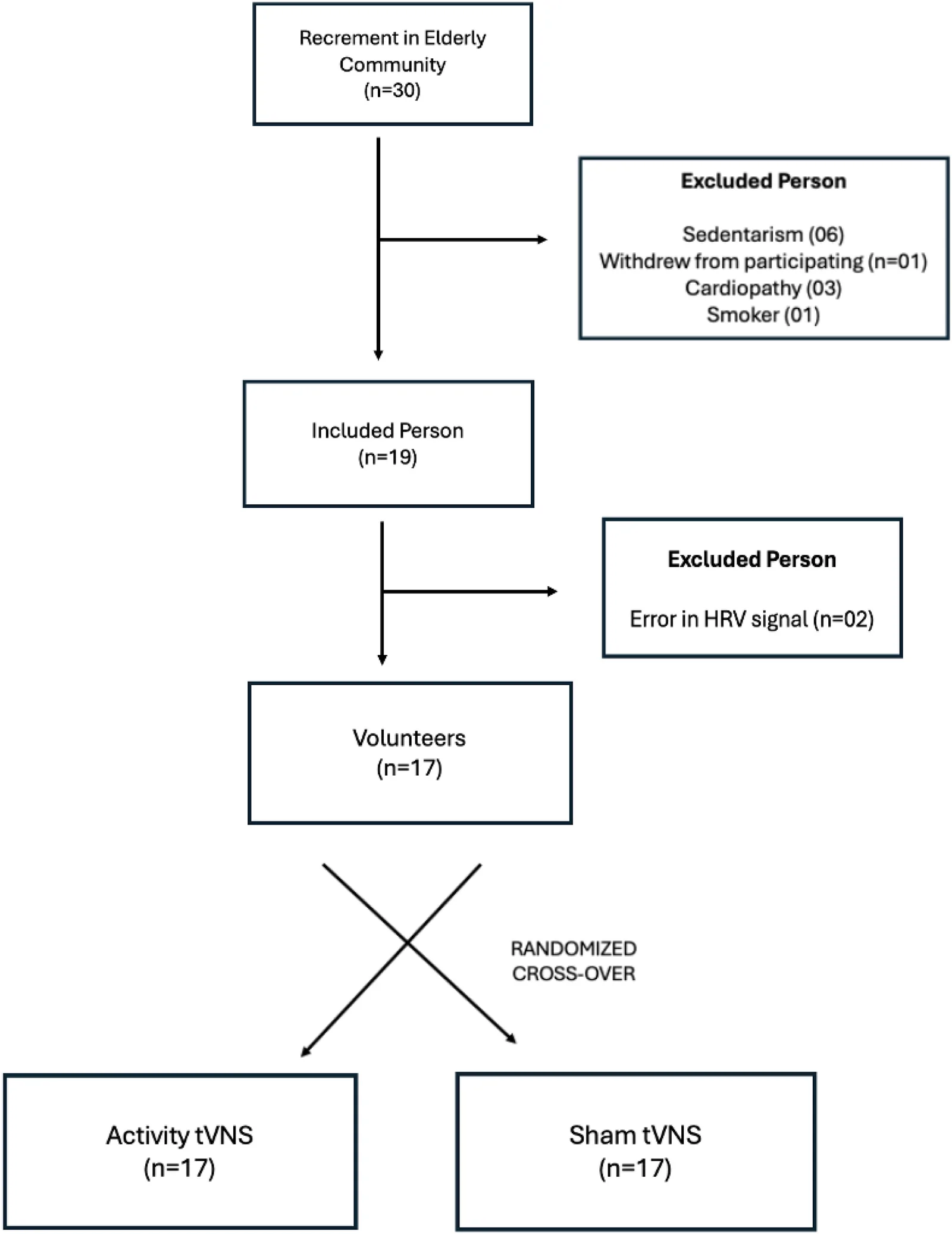
**Study protocol flowchart.** Flow of participants through the study, including randomization, interventions (tVNS and sham), and outcome assessments (HRV and 6MWT).

Outcome measures were collected by trained researchers at baseline and after each intervention session. Measurements and interventions were conducted in a controlled laboratory environment. Analyses of inclusion criteria, obtaining informed consent, data collection, and statistical analyses were performed by researchers who were blind to group allocation. All participants were evaluated and received intervention information in a research laboratory.

The study was conducted according to the ethical principles of the Declaration of Helsinki and approved by the Research Ethics Committee of the Federal University of Amazonas (approval #69,790,623.5.0000.5020). The trial was registered at the Brazilian Clinical Trials Registry (REBEC) #RBR-4dmj9xj.

### Participants and therapists ‒ inclusion and exclusion criteria

Participants were community-dwelling elderly women. Eligibility criteria were as follows:

• Aged 60 to 75 years.
• All were classified as physically active according to the short version of the IPAQ questionnaire.
• Able to walk independently, with or without assistive devices.
• Provide written informed consent.
• Available to attend both intervention sessions with the required washout period.
• Have adequate cognitive function for study participation, screened by the Mini-Mental State Examination (MMSE), with cutoff scores of 26 for highly educated, 18 for those with elementary/middle education, and 13 for illiterate participants.
• Had any unstable cardiovascular disease or other uncontrolled chronic conditions that could interfere with the safety or the conduct and interpretation of testing and training protocols.
• Had implanted electronic devices (e.g., pacemaker) or any contraindication to non-invasive electrical stimulation.
• Had undergone neurosurgical procedures.

Participants were excluded if they:

• Volunteers were excluded if they withdrew from the study or if data capture problems occurred during monitoring.

Therapists who delivered the interventions were eligible if they had received specific training from the research coordinators, who possess >10-years of clinical experience in physical therapy and neuromodulation. Interventions (active tVNS or sham) were prepared and set by a research assistant to ensure therapists were blinded to group allocation.

### Sample size

Because no previous study had evaluated the effects of tVNS on functional capacity in older adults, the initial sample size was estimated from the crossover study by Antonino et al.^7^ To account for the expected greater variability in autonomic responses among older adults, the original sample (n = 13) was doubled, resulting in an initial target of 26 participants. Considering an anticipated 20% attrition rate, the planned recruitment was increased to 30 participants. After enrollment of the first 10 participants, a predefined interim analysis demonstrated a moderate effect size (Cohen's *f* = 0.38) for resting interbeat interval (iRR). Using this effect size, α = 0.05, and power = 80%, the minimum required sample size was recalculated as 17 participants using G*Power software. Therefore, recruitment continued until 20 participants had been enrolled to compensate for possible losses.

### Randomization and initial assessment

Randomization was computer-generated by a researcher not involved in participant recruitment or assessment. For the crossover design, the order of interventions (active tVNS or sham) assigned to each participant was determined in a 1:1 ratio, ensuring balanced allocation. The intervention order for each participant was concealed in sequentially numbered, opaque, and sealed envelopes, prepared before the start of the study by a research assistant not involved in other study procedures.

After baseline assessments were completed, the allocation envelope for each participant was opened by the intervention therapist to determine the sequence of interventions to be received. All outcome measurements were performed by assessors who were blinded to the intervention order and group allocation. Each enrolled participant received a unique identification code to protect confidentiality before, during, and after the trial.

To minimize errors and ensure data reliability, all measurement procedures were reviewed before data collection by the responsible researcher and a trained team composed of SAAS, SDAS, TISF, and FSS. Volunteers who met the inclusion criteria and agreed to participate in the research were provided with information on how the interview would proceed, and subsequently, they were informed of the day and time for the assessment and intervention/sham sessions. If the participant wished, a family member or caregiver could assist them in answering questions and accompany them during appointments. The interview consisted of one day of information collection and three days of evaluation with the administration of tests and implementation of the intervention/sham with crossover, with each session lasting approximately 2-hours. The interview included a sociodemographic questionnaire and a short International Physical Activity Questionnaire (IPAQ). After that, volunteers were randomized through exposure assignment using opaque envelopes. The exposures were performed in a randomly pre-defined manner through drawing, with a twenty-four-hour interval between them. The exposures were labeled as A (Sham intervention) and B (tVNS intervention), as shown in.

### Blinding

The study was characterized as double-blind. For the first blinding, the volunteers receiving the stimulation did not know that the electrical stimulus on the cymba conchae or the earlobe was effective on the parasympathetic system. Therefore, they did not actually know which period was sham or active. The second blinding was applied to the data analysis; the person who performed the analysis did not know which period was sham or stimulus, as letters A and B were used to designate the group names.

### Physical evaluation questionnaire

The level of physical activity was assessed using the short form of the IPAQ, which includes questions on type, frequency, and duration of physical activities performed in a typical week. In total, the questionnaire consists of eight items addressing the number of days and duration of vigorous activities, moderate activities, walking, and sitting time during the week.^8^

### Heart rate variability evaluation

HRV was assessed using a non-invasive and low-cost procedure suitable for various environments, with a minimum recording time of five minutes (or 256 beat-to-beat intervals). A digital heart rate monitor (Polar H10, Polar, Finland), with a sampling rate of 1000 Hz, was used to capture cardiac signals. Data were transmitted via the Elite HRV app (South Carolina, USA) and analyzed with Kubios 3.0 software (Kuopio, Finland).

The analysis included HRV parameters in both the time and frequency domains. Time-domain measures comprised Interbeat Interval (iRR), mean HR, the Standard Deviation of NN intervals (SDNN), and root Mean Square of Successive Differences (rMSSD). Frequency-domain measures included absolute high frequency (HF: 0.04–0.15 Hz), normalized low frequency (LF: 0.15–0.40 Hz), and the LF/HF ratio.^9^

All assessments were performed in a quiet environment with minimal disturbances, under controlled room temperature (20–24 °C). The time of day (morning, afternoon, or evening) was standardized across assessments to account for circadian influences. Before the procedures, participants were instructed to avoid stimulant substances (alcohol, caffeine, nicotine, chocolate, soda, energy drinks), to ensure adequate sleep, and to refrain from vigorous physical activity the day before testing. On the day of assessment, vital signs (blood pressure, respiratory rate, and heart rate) were monitored, and electronic devices were turned off to prevent interference.^10^

Participants rested in the supine position for 10-minutes before and during stimulation. HRV data were recorded continuously throughout the 30-minute intervention, and values from minutes 25–30 were analyzed.

### Functional capacity evaluation

Functional capacity was assessed with the six-Minute Walk Test (6MWT), a submaximal test widely used to evaluate global cardiovascular and muscular responses. Before and after the test, dyspnea and lower limb fatigue were rated using the Borg perceived exertion scale.

The 6MWT was conducted in a 30-meter corridor, free from pedestrian traffic, where participants were instructed to walk as fast as possible for six minutes. The total distance walked was measured once per intervention (tVNS or sham). To minimize learning effects, participants completed a familiarization walk during the first assessment day. The predicted 6MWT distance was calculated using the reference equation proposed by Enright and Sherrill,^11^ which considers age, sex, height, and body weight.

HRV was recorded during the recovery period, from minutes 10 to 15 after the test. Heart Rate Recovery (HRR) was also calculated at 1st- and 5th-minutes post-exercise. All tests were conducted by the same trained examiner.

### Intervention

Participants were placed comfortably in the supine position with head support. The left ear was inspected to ensure the absence of jewelry, skin lesions, or cosmetic products. Both the auricle and earlobe were cleaned with cotton swabs soaked in 70% alcohol. Electrodes were also cleaned and then coated with a thin layer of conductive gel before placement. The electrostimulation device used was EL608 Control Connect (NKL, Santa Catarina, Brazil).

### Transcutaneous auricular Vagus nerve stimulation (tVNS)

In the active stimulation condition, the positive electrode (black) was placed on the outer part of the left ear and the negative electrode (red) on the cymba conchae. Stimulation was delivered at 25 Hz, 500 µs pulse width, in cycles of 30 s on and 60 s off, for a total of 30-minutes. Intensity was adjusted to the sensory threshold, remaining below the pain threshold (as per Fig. 2B).

**Fig. 2:**
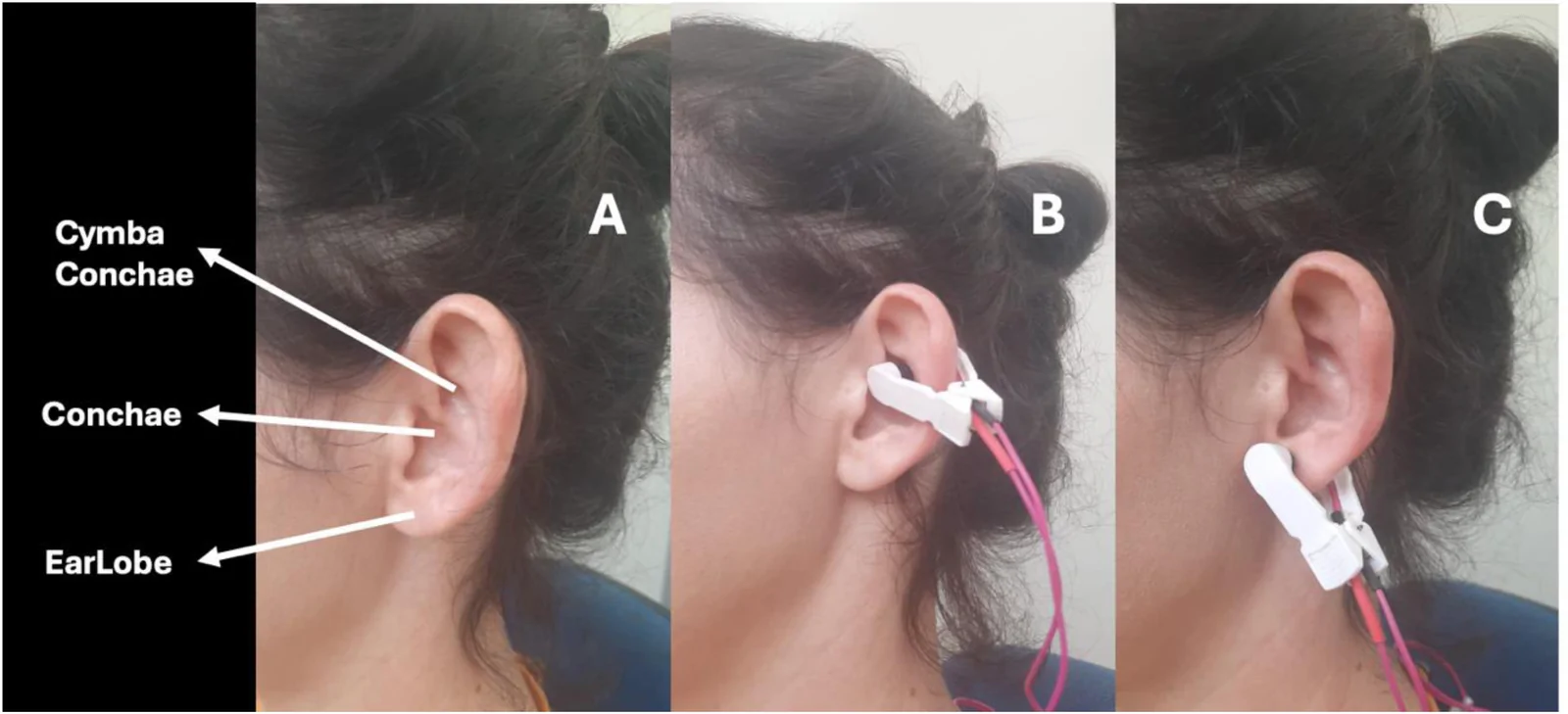
**Electrode placement for active and sham stimulation.** Localization of electrodes during tVNS (cymba conchae) and sham (earlobe) conditions.

### Active sham transcutaneous earlobe stimulation

In the sham condition, the positive electrode (black) was placed on the anterior part of the right earlobe and the negative electrode (red) on the posterior part. The same stimulation parameters were applied (25 Hz, 500 μs, 30 s on/60 s off, intensity below pain threshold) for 30-minutes. At the end of both active and sham sessions, electrodes were removed, the ear was cleaned, and any adverse effects were recorded (as per Fig. 2C).

### Primary outcome

The primary outcome is the distance walked during the 6-Minute Walk Test (6MWT), measured in meters. Participants will be instructed to walk as far as possible for six minutes along a straight corridor, and the total distance covered will be recorded. After a practice trial, the value obtained during a single test will be used for analysis.

### Secondary outcomes

#### Heart rate variability measurement

Heart Rate Variability (HRV) will be measured using a validated heart rate monitor before, during, and after the walking test. Walking step length and cadence will also be assessed during the 6MWT. Step length will be calculated by dividing the distance covered by the number of steps taken and reported in meters. Walking cadence will be determined by dividing the number of steps by the total time (in minutes) of the test, and reported in steps/min.

#### Data monitoring body

An independent researcher, who will be blind to the group allocations, will monitor any adverse effects and perform database management and statistical analyses. The treating therapists will be responsible for the monitoring of doses and compliance.

### Statistical analysis

For statistical analysis, the data will be expressed as mean and standard deviation for normally distributed data, median and interquartile range for non-normally distributed data, and percentage, minimum, and maximum values when necessary. Additionally, data on the difference between means and medians, Confidence Interval of 95% (CI_95%_), and effect size will be provided. Initially, the Shapiro-Wilk and Levene tests will be used to assess the normality and homogeneity of continuous variables, respectively. For paired analysis between intervention and sham moments, the paired t student test or Wilcoxon test were employed. When analyzing variables of time and moment in paired analysis, an ANOVA two-way (within-subjects) post hoc Bonferroni tests were used. A statistical difference was considered significant at p < 0.05, and statistical analyses were performed using GraphPad Prism 8.0 (GraphPad, California, USA), Jamovi 2.3 (Jamovi Project, Sydney, Australia), and G*Power 3.1 (University of Dusseldorf, Dusseldorf, Germany).

## Results

In total, 31 community-dwelling elderly women were initially deemed eligible to participate in the study. After the application of the established inclusion and exclusion criteria, 20 participants were selected. During the testing phase, signal acquisition issues from the heart rate monitor affected three volunteers, impeding the analysis of their biological signals. Consequently, the final sample comprised 17 participants.

### Sample characteristics

The mean age of the participants was 67±3.9-years, with a mean Body Mass Index (BMI) of 29±4.6 kg/m^2^. Hypertension was the most prevalent comorbidity (65%), followed by prior COVID-19 infection (53%) and depressive disorder (24%). The majority reported use of antihypertensive medications, mainly AT1 receptor blockers (35%) and diuretics (35%). Table 1 summarizes the anthropometric, clinical, and educational characteristics of the sample.

**Table 1:** Baseline characteristics of the study participants (n = 17).

**N=17**	
**Age (Years)**	67 ± 3.9
**BMI (kg.m^2^)**	29 ± 4.6
**Physical Activity (%)**	100
**Comorbidities (%)**	
Hypertension	65
Hypercholesterolemia	24
Diabetes	12
Depression/Anxiety	24
Osteoporosis	12
Hypothyroidism	0.5
COVID-19	53
**Medication (%)**	
Beta blocker	0
AT1 blocker	35
Diuretics	35
ACE inhibitor	29
Statin	24
SAA	12
Anxiolytic/Ant depressive	5
**Education (%)**	
Any	21
Incomplete Elementary School	58
complete Elementary School	5.3
Completed Higher School	5.3
Completed Higher Education	11

Anthropometric, clinical, and sociodemographic characteristics of community-dwelling older women included in the study. Values are expressed as mean ± SD or n (%). BMI, Body Mass Index; MMSE, Mini-Mental State Examination; IPAQ, International Physical Activity Questionnaire; ACE inhibitors, Angiotensin-Converting Enzyme inhibitors; AT1 blockers, Angiotensin II receptor blockers; ASA, Acetylsalicylic Acid; SAA, Selective Serotonin Reuptake Inhibitors (SSRIs), anxiolytics, and antidepressants.

### Autonomic function at rest

Analysis of HRV parameters revealed significant differences between tVNS and sham conditions at rest. Compared to baseline, tVNS significantly increased the interbeat interval (iRR; p = 0.008, small effect size, Cohen’s *F* = 0.20) and reduced heart rate (HR; p = 0.03, large effect size, Cohen’s *F* = 0.45). No significant differences were observed in parasympathetic markers such as rMSSD and absolute HF. However, tVNS led to a reduction in sympathetic activity, with lower LF (p = 0.049) and LF/HF ratio (p = 0.028), both showing moderate effect sizes (point-biserial correlation = 0.55 and 0.65, respectively) (Fig. 3).

**Fig. 3:**
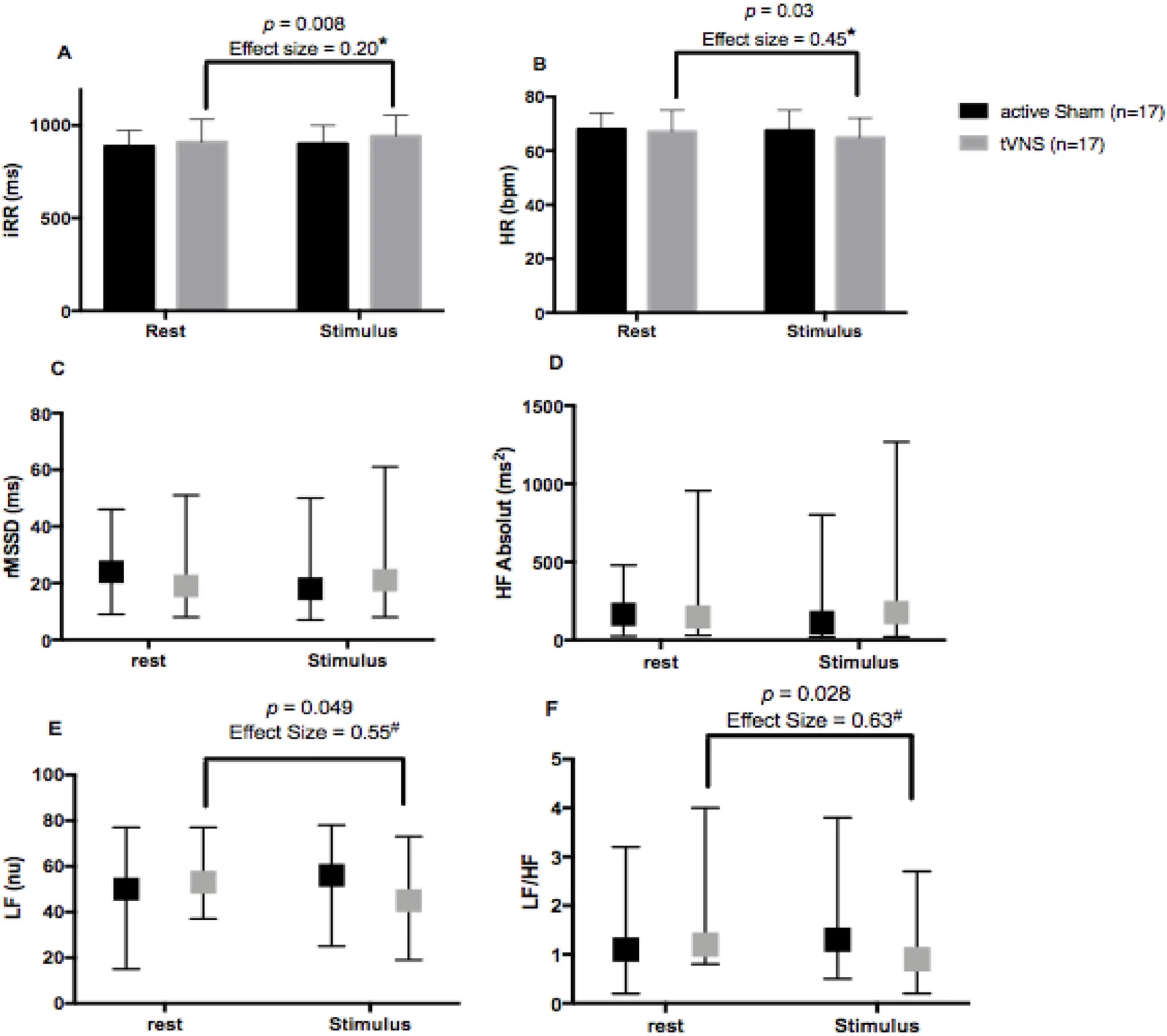
**Autonomic function at rest during tVNS and sham conditions.** (A) Interbeat interval (iRR). (B) Heart Rate (HR). (C) rMSSD. (D) High Frequency (HF). (E) Low Frequency (LF). (F) LF/HF ratio. Data are shown as mean ± SD or median [IQR]. *p < 0.05 between conditions.

### Functional capacity and clinical variables

No significant differences were observed between tVNS and sham for resting SBP (p = 0.66), DBP (p = 0.71), HR (p = 0.38), SpO_2_ (p = 0.25), or Borg score (p = 0.18). Similarly, recovery values for SBP, DBP, HR, SpO_2_, and Borg at the end of the 6MWT did not differ significantly between conditions. Regarding functional capacity, the difference in absolute walking distance did not reach statistical significance (p = 0.07), the observed effect size (Cohen's *d* = 0.43) suggests a moderate practical effect favoring active tVNS. However, when expressed as a percentage of predicted distance, participants performed significantly better in the tVNS condition (100±15%) compared to sham (93±14%), with a moderate effect size (Cohen’s *d* = 0.65; p < 0.01; mean difference = −7.2%, 95% CI: −12.4 to −1.9) (Table 2)

**Table 2:** Clinical and functional variables during rest and recovery after the six-minute walk test.

	**Sham tVNS**	**Real tVNS**	**Diff values**	**CI_95%_**	**Valor-p**	Effect Size*
**Rest**						
SBP (mmHg)	122 ± 14	123 ± 16	−2.4	4.0 – −6.1	0.66	0.10
DBP (mmHg)	77 ± 9	79 ± 13	−0.8	5.3 – −3.7	0.71	0.08
HR (bpm)	74 ± 13	77 ± 14	−2.8	3.8 – −9.5	0.38	0.20
Borg	0 [0 – 3]	0 [0 – 2]	**‒**	**‒**	0.18	‒
SpO_2_ (%)	96 ± 1.7	96 ± 1.8	0.9	2.0 – - 0.18	0.25	0.40
**Recovery**						
SBP (mmHg)	139 ± 22	134 ± 18	5.1	19 – −8.7	0.45	0.17
DBP (mmHg)	84 ± 10	83 ± 12	1.32	3.2 – −5.8	0.55	0.14
HR (bpm)	101 ± 19	104 ± 18	3.1	2.8 – −9.0	0.28	0.25
Borg	2 [1 – 4]	2 [0 – 4]	**‒**	**‒**	0.06	**‒**
SpO_2_ (%)	97 ± 1.5	97 ± 1.6	0.63	0.4 – −2.2	0.17	0.32
Distance Walked (m)	422 ± 66	442 ± 57	−20.4	2.0 – - 42.8	0.07	0.43
Distance Walked Predicted (%)	**93±14**	**100±15**	**−7.2**	**−12.4 – −1.90**	**0.01**	**0.65**

Comparison between tVNS and sham conditions. Values are expressed as mean ± SD or median [IQR]. iRR, inter-beat interval; rMSSD, Root Mean Square of Successive Differences; LF/HF, Low-Frequency/High-Frequency ratio; SBP, Systolic Blood Pressure; DBP, Diastolic Blood Pressure; SpO₂, Peripheral Oxygen saturation; Borg, Borg Rating of Perceived Exertion. Negative values indicate improvement favoring active tVNS.

### Heart rate recovery (HRR) after 6MWT

There was no significant association between tVNS and HRR at the first- or fifth-minutes post-exercise (Fig. 4).

**Fig. 4:**
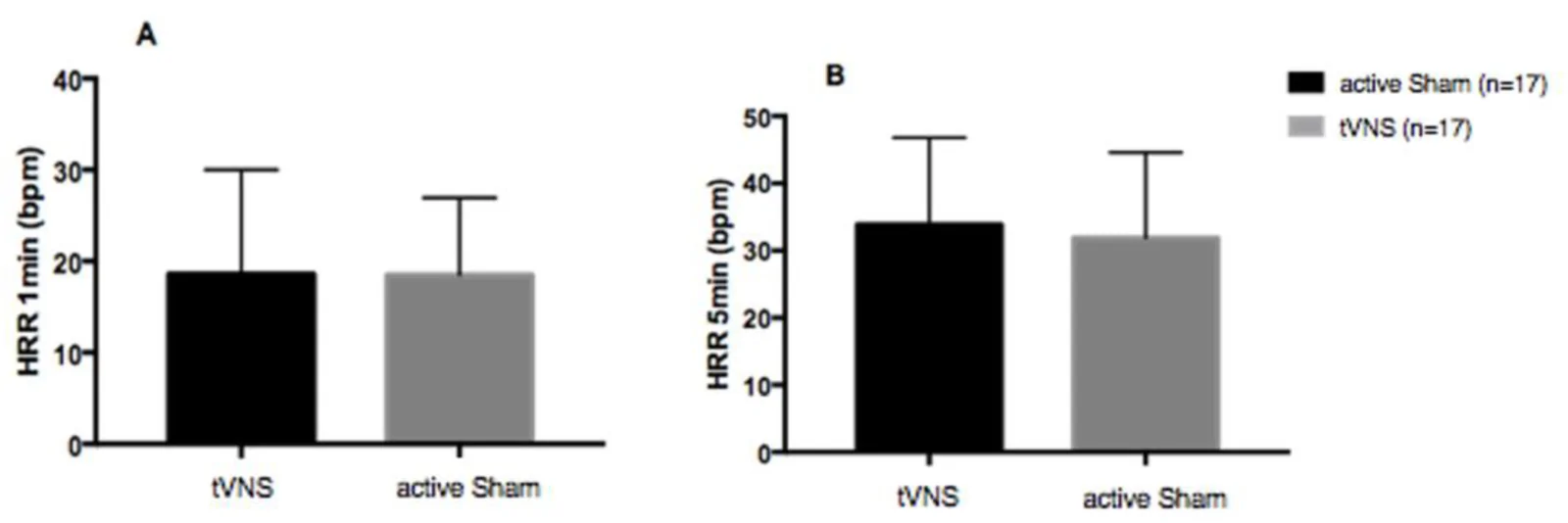
**Heart rate recovery (HRR) after 6MWT.** HRR at the first- and fifth-minutes post-exercise under tVNS and sham conditions. Data presented as mean ± SD.

No adverse events related to either active or sham stimulation were observed during the study.

## Discussion

This is the first study to demonstrate the effect of tVNS on the functional capacity of community-dwelling elderly. The participants received either stimulation or sham in a randomized manner on different days. It was shown that the stimulation group experienced increased parasympathetic activity during stimulation, and after stimulation, participants walked a greater predicted distance compared to the sham group.

Although absolute walking distance did not significantly increase after a single tVNS session, participants achieved a significantly greater percentage of their predicted walking capacity. Clinically, this finding may indicate that participants performed closer to their expected physiological potential rather than simply walking farther. Such improvement may reflect enhanced movement efficiency, subtle optimization of pacing strategy, or reduced physiological cost during walking, effects that may precede measurable changes in absolute walking distance. Because the participants were physically active, community-dwelling older women with relatively preserved functional capacity, a ceiling effect may also have limited further gains in the total distance covered during a single acute intervention.

### Motor effect of vagal stimulation

Some studies have attempted to identify the impact of the ascending vagal system on the motor system. Capone et al.^6^ evaluated 10 volunteers who underwent stimulation for 60-minutes, which influenced the cardiovascular system, with an increase in the motor threshold, short interval intracortical inhibition, and increased GABA activity. Ven Midden (2023), in a double-blind study with a larger sample size (30 volunteers) and with sham and intervention groups, demonstrated the same results as the Capone et al. (2015), in which tVNS increased the parameters of cranial transmagnetic stimulation in the motor area, with a reduction in short-interval intracortical inhibition, and this effect is caused by increased cortical GABAergic system activity. On the other hand, Yun et al.^12^ demonstrated that the effect of tVNS for 30-minutes of stimulation in young adults shows differences in excitability response, and that some volunteers are not capable of being influenced by the stimulation.

Enhanced cholinergic activity may also contribute to improved motor performance by facilitating cortical motor network activation, improving sensorimotor integration, and optimizing motor planning. Together with the observed increase in parasympathetic modulation, these mechanisms may partially explain why participants performed closer to their predicted functional capacity despite the absence of a statistically significant increase in absolute walking distance.

In older and middle-aged volunteers (mean age 59.91±6.87 years), Gerges et al.^13^ demonstrated that the same effect is also observed in these participants. The authors evaluated the effect at different stimulation durations (30-minutes and 60-minutes). The authors found greater effects after 60-minutes of stimulation; however, cortical stimulation was observed at both times. In the current study, the protocol used was 30-minutes, and even so, it was possible to observe effects on autonomic behavior and functional capacity. Although cortical activity was not evaluated in these volunteers, the response in functional capacity is a very important factor for clinically assessing the aspects related to tVNS.

In addition to the GABAergic pathway, Horinouchi et al.^14^ demonstrated that the increase in excitability may be directly related to an enhancement of the cholinergic pathway. One difference compared to other methodologies was that the stimulated volunteers received 15- and 30-minutes of stimulation, and from 15-minutes onward, it was possible to observe an increase in short-latency afferent inhibition, which is indirectly related to the cholinergic system.

Still regarding the effect of tVNS on motor capacity, Ackland et al.^15^ conducted a randomized, crossover clinical trial involving young, healthy volunteers who underwent 7-days of bilateral tVNS. The intervention resulted in a significant increase in physical capacity, with a 1.04 mL/kg/min improvement in peak oxygen uptake during the stimulation phase compared to sham. Notably, these gains were accompanied by reductions in both chronotropic response and respiratory rate at peak exertion. Such findings may provide a valuable reference point for interpreting our results in an elderly cohort of apparently healthy and physically active participants.

Future studies are needed to analyze the effect of longer stimulation times. Additionally, it is important to observe motor behavior after a double-blind, controlled study (the current study is cross-over). Using the effect size from our study, we calculated the sample size for a study with two groups. Using a Cohen’s *f* effect size = 0.3, α = 0.05, 1-β = 0.95, two groups, and two time points for two-way ANOVA, 40 participants are required for greater robustness and to confirm the results with a more powerful methodology than ours (software used: G*Power 3.1, University of Düsseldorf, Düsseldorf, Germany]).

### Vagal stimulation in motor rehabilitation

Dawson et al.^16^ conducted one of the most elegant studies on vagal stimulation in neurological rehabilitation in patients with upper limb motor deficits after stroke. The study, which was published in the world’s leading medical journal, The Lancet, demonstrated that invasive vagal stimulation was able to improve upper limb movement capacity compared to the control group. In addition, a sub-analysis of the main study was able to assist in the recovery of young individuals with less severe injuries.^16^

Wu et al.^17^ conducted a pilot study showing that tVNS can increase the functional capacity of the upper limb in volunteers with post-stroke sequelae. Furthermore, another pilot study demonstrated that tVNS can increase upper limb capacity.^18^ All these results are in line with the studies by Dawson group.

The current study is also a cross-over design and was conducted with community-dwelling older adults without motor diseases. However, it opens a new opportunity for further research in frail older adults, aiming to facilitate motor gains and prevent motor decline with age or before the onset of more severe neurological diseases, such as Parkinson’s Disease (PD).

Studies involving PD have also shown a motor effect in the rehabilitation of these patients. Zhang et al.,^19^ in a pilot study, demonstrated that tVNS improves gait and increases brain activity in patients with PD. The authors reported improvements in both gait and balance capacity. Marano et al.^20^ showed that the possible mechanism for gait improvement in PD is the increased activity in the subthalamic nucleus, which has great functional capacity for these volunteers. Furthermore, these results may help to understand other non-cortical effects of tVNS in the study volunteers.

### Limitations

Additional limitations should also be acknowledged. First, cortical excitability was not directly assessed using neurophysiological techniques such as transcranial magnetic stimulation, preventing confirmation of the proposed neuromodulatory mechanisms. Second, although medication use reflected the characteristics of this age group, antihypertensive agents and statins may have influenced autonomic regulation.

## Conclusion

This pioneering randomized crossover study provides preliminary evidence that acute transcutaneous vagus nerve stimulation enhances parasympathetic modulation and improves performance relative to predicted functional walking capacity in healthy older women. However, the primary outcome, absolute 6-minute walk distance, did not reach statistical significance. Therefore, these findings should be interpreted as hypothesis-generating and warrant confirmation in adequately powered randomized clinical trials evaluating repeated stimulation protocols and clinically meaningful outcomes.

## Declaration of generative AI

During the preparation of this work, the authors did not use generative artificial intelligence or AI-assisted technologies in the writing of this manuscript.

## Authors’ contributions

SAAS: Investigation; Data curation; Writing-original draft. FZSA: Conceptualization; Methodology; Supervision; Writing-review & editing. SDAS: Investigation; Formal analysis. TISF: Investigation; Resources. MPD: Investigation; Validation. FSS: Investigation; Data Curation. LDO: Investigation; Resources. CLG: Formal analysis; Writing-review & editing. FFAV: Methodology; Validation; Writing-review & editing. GPTA: Conceptualization; Project administration; Supervision; Writing-review & editing.

## Declaration of competing interest

The authors declare that there are no conflicts of interest regarding the publication of this manuscript.

## Data Availability

All relevant data are available within the manuscript.
